# The estimands framework: a primer on the ICH E9(R1) addendum

**DOI:** 10.1136/bmj-2023-076316

**Published:** 2024-01-23

**Authors:** Brennan C Kahan, Joanna Hindley, Mark Edwards, Suzie Cro, Tim P Morris

**Affiliations:** 1 MRC Clinical Trials Unit at UCL, University College London, London WC1V 6LJ, UK; 2 Department of Anaesthesia, University Hospital Southampton NHS Foundation Trust, Southampton, UK; 3 Southampton NIHR Biomedical Research Centre, University of Southampton, Southampton, UK; 4 Imperial Clinical Trials Unit, School of Public Health, Imperial College London, London, UK

## Abstract

Estimands can be used in studies of healthcare interventions to clarify the interpretation of treatment effects. The addendum to the ICH E9 harmonised guideline on statistical principles for clinical trials (ICH E9(R1)) describes a framework for using estimands as part of a study. This paper provides an overview of the estimands framework, as outlined in the addendum, with the aim of explaining why estimands are beneficial; clarifying the terminology being used; and providing practical guidance on using estimands to decide the appropriate study design, data collection, and estimation methods. This article illustrates how to use the estimands framework by applying it to an ongoing trial in emergency bowel surgery. Estimands can be a useful way of clarifying the exact research question being evaluated in a study, both to avoid misinterpretation and to ensure that study methods are aligned to the overall study objectives.

Research studies are often used to answer questions about the effect of an intervention. However, deciding on the precise research question to ask, or how best to design the study to answer that question, can be challenging. Consider the FLO-ELA trial, a pragmatic trial comparing two methods of fluid delivery (cardiac output monitor *v* clinician judgment) in patients undergoing emergency bowel surgery.^1^ Because of the lead-in time required to prepare the intervention, a small delay between randomisation and the start of surgery is necessary, and so some participants in FLO-ELA could have their surgery cancelled after randomisation, either because they become too unwell or the underlying issue has resolved itself.

A standard approach for pragmatic trials is to conduct an intention-to-treat analysis, which would include participants who did not undergo surgery.^2^ However, consideration of the research question could lead investigators to question this approach. An intention-to-treat analysis answers the question “What is the difference between fluid delivery methods, regardless of whether patients undergo surgery?” Because fluid is only given to patients who do undergo surgery, interest would typically lie in the effect in these patients. Thus, a more relevant research question might be “What is the difference between fluid delivery methods, in patients who actually undergo surgery?”^1^ ^3^ ^4^ Having settled on the most relevant question, investigators can then identify a more appropriate method of analysis to answer this question.^3^

Here, cancellation of surgery is an example of an intercurrent event, which includes events that affect a patient’s assigned treatment (for instance, if they stop taking treatment early, or receive a different treatment to the one they were meant to).^5^ It is important to consider how such intercurrent events are reflected in the research question, because different ways of doing so can affect interpretation of results (box 1). For instance, in FLO-ELA, the intervention cannot have an effect in patients not undergoing surgery, and thus inclusion of these patients pulls the overall treatment effect towards zero, rendering it more difficult to identify a beneficial (or harmful) intervention effect.^3^ ^4^

Box 1 **Importance of intercurrent events**

**Example 1**

In a study of dupilumab versus placebo for uncontrolled asthma, patients in the placebo arm might receive rescue treatment more often than patients in the dupilumab arm.^6^ Where does interest lie: in the effect of dupilumab versus placebo when rescue forms part of the two treatment strategies, or in the effect of dupilumab if patients had not received rescue?

**Example 2**

In a study comparing two different fluid delivery methods in patients undergoing emergency bowel surgery, patients could have their surgery cancelled after enrolment.^1^ Do researchers want to compare the two fluid delivery methods only in those patients who actually undergo surgery, or in all patients regardless of whether they undergo surgery?

**Example 3**

In a study evaluating a music intervention delivered by caregivers for people with dementia on symptom reduction at 90 days, some participants could die before day 90.^7^ Should researchers use their final symptom score before they died to evaluate the intervention effect while they still lived, or assign their 90 day score a low value, to reflect that death is a poor outcome?

**Example 4**

In a study of triamcinolone versus usual care in patients undergoing eye surgery, some patients might take additional non-study treatments.^8^ Should researchers evaluate the effect of triamcinolone alongside these additional non-study treatments, or its effect if patients had not taken any additional treatments?

Estimands provide a way to clarify research questions (box 2).^4^ ^5^ ^14^ ^15^ ^16^ ^17^ ^18^ ^19^ ^20^ ^21^ ^22^ ^23^ ^24^ ^25^ ^26^ ^27^ The addendum to the ICH E9 harmonised guideline on statistical principles for clinical trials (ICH E9(R1)) describes a framework for incorporating estimands into a study’s design. In this paper, we summarise the estimands framework, as outlined in the ICH E9(R1) addendum,^5^ with the aim to explain why estimands are beneficial; clarify the terminology being used; and provide practical guidance on using estimands to decide the appropriate study design, data collection, and estimation methods. Box 3 provides a list of key terms.

Box 2 **How estimands can clarify research questions**

- It is important to understand which type of treatment effect a study sets out to estimate. Historically, two types of studies have been considered^9^: pragmatic studies that seek to estimate an intervention’s real world effect, and explanatory studies that seek to estimate an intervention’s effect under ideal conditions.
- However, these two paradigms are not sufficient to precisely define the exact research question, because within these broad definitions exist multiple versions of a pragmatic or explanatory effect that could be estimated. Thus, international guidelines have called for greater clarity.^5^
- Estimands extend the commonly used PICO (population, intervention, comparator, outcome) framework for defining research questions by adding two additional attributes: the summary measure, which defines how outcomes are summarised and compared between treatments; and the strategies used to handle each type of intercurrent event, which define how things such as treatment switching or treatment discontinuation are handled in the treatment effect definition.
- Estimands are now required in some reporting guidelines,^10^ ^11^ ^12^ and medicine regulators in Europe, US, Canada, Singapore, China, Switzerland, and Chinese Taipei now require regulatory applications to include estimands, while regulators in Brazil, the Republic of Korea, and Japan are currently in the process of implementing the inclusion of estimands.^13^

Box 3 **List of key terms**

Estimand: A description of the exact treatment effect a study aims to quantify.
Estimator: The statistical method used to compute the estimate of the treatment effect.
Estimate: The numerical value computed by the estimator. For example, in a study reporting an estimated mean difference between groups of −0.7 (95% confidence interval −0.3 to −1.1), the value −0.7 is the estimate.
Sensitivity analysis: Analyses designed to explore the robustness of the main results from deviations from the estimator’s underlying assumptions. Sensitivity analyses target the same estimand as the main estimator, using different plausible assumptions.
Intercurrent events: Post-baseline events (post-randomisation events in randomised trials) that affect either the interpretation of outcome data (eg, treatment non-adherence or use of rescue treatment) or the existence of outcome data (eg, death if not already used as part of the outcome definition). Missing data or loss to follow-up are not intercurrent events.

**Summary points**

Estimands provide a structured description of the treatment effect(s) a study intends to quantify
Their use helps to align a study’s methods with its aims and ensures clarity in the treatment effect’s interpretation
The study design, data collection, and analysis methods can all affect the ability to estimate the desired estimand(s), and thus should be chosen with the estimand(s) in mind
Estimands should be routinely reported to ensure clarity of the research question, and facilitate critical appraisal of the study’s methods

## The estimands framework

An estimand describes the treatment effect a study sets out to quantify, and use of estimands can help to both clarify the research questions being investigated (table 1) and ensure that appropriate study methods are used to answer these questions. The estimands framework is a way of incorporating estimands into a study to ensure these goals are met (table 2).

**Table 1:** Example of how estimands can help researchers understand the research question

Study description	Statistical methods	Problems understanding the research question	How estimands explain the research question
A trial compared dupilumab with placebo on forced expiratory volume (FEV_1_) at week 12 in patients with uncontrolled persistent asthma. Some patients stopped dupilumab early or received rescue treatments for exacerbations.	Data were analysed on an intention-to-treat basis. Outcome data after receipt of rescue treatment or discontinuation of dupilumab was treated as missing, and a mixed model for repeated measures was used to estimate the treatment effect.*	Because the statistical methods do not make explicit how the research question handles early stopping of dupilumab or receipt of rescue treatment, readers must infer this.* Since the analysis was by intention to treat, they might incorrectly assume that interest lies in the effect of dupilumab regardless of the early stopping or use of rescue treatment.	The estimand explicitly describes how early stopping and receipt of rescue treatment are handled in the research question: “The estimand is the difference in the mean FEV_1_ at week 12 between dupilumab plus standard of care versus placebo plus standard of care, in patients with uncontrolled persistent asthma, if they were to continue using dupilumab over the entire trial period without the use of rescue treatment.”

* In this setting, the mixed model for repeated measures estimates dupilumab’s hypothetical effect if patients were to continue taking dupilumab and did not receive rescue treatment, because investigators treated outcome data after receipt of rescue treatment or discontinuation of dupilumab as missing. Here, the mixed model served to implicitly impute what the outcome data would have been had participants not received rescue treatment or discontinued. Here, deciphering the research question requires an in-depth understanding of the mechanics underlying mixed models for repeated measures, which not all readers will have.

**Table 2:** The estimands framework, using the ASCOT trial^8^ as an example

Steps	Example from the ASCOT trial*	Explanation
1) Define the estimand for each study outcome based on the study’s objective	The primary estimand is the difference in the proportion of patients with an improvement on the ETDRS letter score between baseline to six months of at least 10 points between triamcinolone during standard surgery versus standard surgery alone, regardless of treatment crossovers or use of any non-study treatments, in patients undergoing vitreoretinal surgery after open globe trauma.	This step helps to ensure that the research question is clearly defined. In the ASCOT trial, the estimand alerts readers to the fact that interest lies in the effect of triamcinolone, regardless of treatment crossovers or use of non-study treatments.
2) Choose the study design, data collection, and statistical methods to enable estimation of the chosen estimands	Consideration of the research question indicates that outcome data should be collected for all patients, regardless of whether patients adhere to their allocated treatments or not; and that all patients with available outcome data must be included in the analysis, regardless of whether they adhere or not.	This step ensures that the study will be able to answer each question it has set out to. In the ASCOT trial, collection of outcome data after non-adherence, and inclusion of all patients in the analysis is necessary to estimate the effect of triamcinolone, regardless of treatment crossovers or use of non-study treatments. The trial found that for this specific research question, triamcinolone had little effect (difference 3.5% (95% confidence interval −8.6% to 15.6%), P=0.91).
3) Perform sensitivity analyses to evaluate the robustness of results to departures from the assumptions underpinning the statistical analyses	Because outcome data were not available for all patients, sensitivity analyses were used to explore whether differing assumptions about the missing data could have affected conclusions. Investigators found that conclusions did not change under the sensitivity analyses.	This step is used to provide assurance as to how reliable study results are. Sensitivity analyses did not change conclusions in the ASCOT trial, which gives readers more confidence that results are correct.

* Some study aspects have been modified for simplicity.

The estimands framework described here was first outlined in the ICH E9(R1) addendum.^5^ However, most aspects of the framework (including the concept of estimands, sensitivity analyses, and ensuring that statistical analyses answer clinically relevant questions) have been acknowledged as being important for years (eg, in the National Research Council’s 2010 study on the Prevention and Treatment of Missing Data in Clinical Trials, as well as in the causal inference literature).^28^ ^29^ ^30^ ^31^ ^32^ ^33^ ^34^ ^35^ ^36^ The estimands framework brings these different concepts together under one general framework, and provides a structured way of approaching each element using common language to describe the concepts.

In the following sections, we describe each aspect of the estimands framework, including what attributes comprise an estimand, general points to consider when choosing a strategy to handle intercurrent events, as well as strategies for implementing the estimands framework.

## What is an estimand?

The term “estimand” is used to specify the research question a study aims to quantify, and thus is widely used across different disciplines, from descriptive epidemiology to prognostic modelling.^37^ ^38^ Here, we describe estimands in the context of studies used to evaluate healthcare interventions.

In this setting, estimands describe the treatment effect the study sets out to quantify for a given outcome. They do so using a structured approach, with standardised terminology. The structured approach ensures that all aspects of the treatment effect are described, while the use of standardised terminology ensures that the estimand can be easily understood.^20^ Importantly, estimands describe a causal effect of treatment—that is, they describe how outcomes would change between different treatment strategies for the same set of participants.^5^ ^36^ ^39^ A separate estimand is defined for each study outcome, although for some outcomes more than one estimand might be of interest. Table 3 lists the five core attributes that comprise an estimand: population, treatment conditions, endpoint, summary measure, and the strategies used to handle each type of intercurrent event in the treatment effect definition.

**Table 3:** Core attributes of estimands

Attribute	Definition	Example from the FLO-ELA trial^1^
Population	Patients for whom researchers want to estimate the treatment effect	Patients ≥50 years old who would undergo emergency bowel surgery under any treatment assignment
Treatment conditions	Different intervention strategies being compared in the treatment effect definition	Intervention group: assignment to protocolised, cardiac output guided, haemodynamic treatment during surgery and for six hours after, regardless of whether cardiac output monitor is followed correctly; usual care group: assignment to intravenous fluid use without cardiac output monitoring or protocol during surgery, and for six hours after
Endpoint	Outcome for each participant that is used in the treatment effect definition	Number of days alive and out of hospital within 90 days of randomisation
Summary measure	Method used to summarise and compare the endpoint between treatment conditions (eg, risk ratio, odds ratio)	Ratio of means
Handling of intercurrent events	Strategies used to handle each intercurrent event* in the treatment effect definition; different strategies could be used for different types of intercurrent events	Surgery cancelled after randomisation (applies to both treatment groups): principal stratum (subpopulation of patients who would undergo surgery under either treatment assignment); receipt of cardiac output monitoring (usual care group): treatment policy; failure to initiate cardiac output monitoring (intervention group): treatment policy; cardiac output monitoring algorithm not followed (intervention group): treatment policy

* Intercurrent events are post-baseline events (or post-randomisation events in randomised trials) that affect the interpretation or existence of outcome data. These events frequently affect receipt of treatment (eg, treatment switching or treatment discontinuation) or preclude existence of the outcome (eg, death, if it is not defined as part of the outcome).

## Intercurrent events

**Fig 1:**
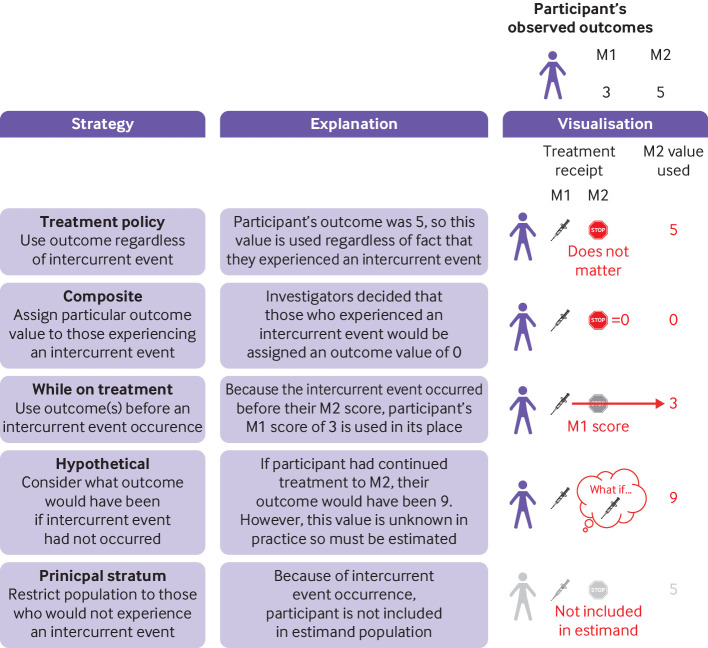
Different strategies regarding intercurrent events. In this example, a randomised trial compares intervention with control to understand how outcomes differ at month 2. However, one participant stops treatment before month 2 (ie, an intercurrent event). The figure shows what happens to this participant under each intercurrent event strategy. Under a composite strategy, investigators have decided to assign a score of 0 to any participant who experienced an intercurrent event. Under a while-on-treatment strategy, because the participant experienced an intercurrent event before month 2, their month 1 score of 3 is used in place of their month 2 score. Under a hypothetical strategy, the participant’s outcome that would have occurred had they continued treatment at month 2 is used (here, it is a value of 9); but in practice, this value will not be known and so must be estimated. M=month

Intercurrent events are post-baseline events (or post-randomisation events in randomised trials) that affect either the interpretation or existence of outcome data (fig 1, box 5). These generally fall into two distinct categories: treatment-modifying events and truncating events. Other types of intercurrent events can also be defined,^5^ but their use is less frequent and we do not consider them here.

Box 5 **Intercurrent events, protocol deviations, and missing data**

The definition of an intercurrent event is broad, encompassing several distinct concepts (eg, treatment-modifying events, truncating events). Owing to some overlap with other common concepts, understanding what is (and what is not) an intercurrent event can be challenging. We summarise below how intercurrent events differ from protocol deviations and missing data.

**Protocol deviations**

Some but not all protocol deviations can also be intercurrent events. Intercurrent event status depends on whether the protocol deviation affects assigned treatment. If it does affect assigned treatment (eg, receipt of prohibited drug treatment), the deviation is also an intercurrent event; if it does not (eg, failure to take proper informed consent), the deviation usually is not an intercurrent event.

Similarly, some but not all intercurrent events can also be protocol deviations. Protocol deviation status will depend on whether the intercurrent event is allowed by the protocol. For instance, if the protocol allows patients to modify or stop treatment in response to an adverse event, this event is not a deviation. However, if a participant receives drug treatment prohibited by the protocol, this event is a deviation.

**Missing data**

Loss to follow-up, study withdrawal, and missing data frequently occur alongside certain intercurrent events, but they are not themselves intercurrent events.^5^ For instance, participants who stop treatment early might also withdraw from the study. However, it is the treatment discontinuation that affects our interpretation of outcome data, and not the withdrawal from the study (which simply poses a missing data issue that needs to be handled as part of the statistical analysis, but not as part of the estimand definition).

Treatment-modifying events affect receipt of the assigned treatment. In the example asthma study described in table 1, early discontinuation of dupilumab and use of rescue treatment are treatment-modifying intercurrent events. Other examples might be if patients received the wrong dose of dupilumab, or if patients in the placebo arm received dupilumab instead. These events affect the interpretation of outcome data because outcomes from participants who experienced the intercurrent event might provide different information about treatment than outcomes from participants who did not experience the intercurrent event.

Truncating events preclude the existence of the outcome. The most common truncating event is death (often referred to as truncation by death). For instance, in the example asthma study, if a patient died at week 6, then their forced expiratory volume (FEV_1_) measurement at week 12 would not exist. Importantly, the week 12 FEV_1_ measurement is not considered to be missing data, which implies that it could have been collected but was not. Other truncating events might be amputation of a limb when the outcome is a symptom score based on that limb, or miscarriage when the outcome is neonatal birth weight. In time-to-event settings, truncating events that prevent the outcome of interest from occurring are often referred to as competing events.

## Strategies to handle intercurrent events in the estimand definition

A strategy to handle each type of anticipated intercurrent event must be defined as part of the estimand. Not all potential events will be relevant for all studies, and so investigators must carefully think through the types of intercurrent event that might occur in their study and consider the different strategies to handle such events (table 4). Intercurrent event strategies must be defined by event rather than by study (ie, different strategies can be used for different types of intercurrent events in the same study). Below, we outline the different strategies that can be used.

**Table 4:** Strategies to handle intercurrent events in the estimand definition

Strategy	Definition	Points to consider
Treatment policy	The intercurrent event is considered part of the treatment strategy, so outcomes are used whether or not the intercurrent event occurred	Cannot be used for truncating intercurrent events, such as death
		Can be used to evaluate the intervention if used as part of routine practice, provided that the intercurrent event under consideration would occur in routine practice as well as in the study setting
Composite	The intercurrent event is incorporated into the outcome definition, and participants who experience the intercurrent event are assigned to a particular outcome value	Modifies the endpoint attribute of the estimand
		Changes the interpretation of the estimand to include the effect of treatment on the occurrence of the intercurrent event
		Different composite estimands could be defined on the basis of the choice of value assigned to the outcome
		Should not be used for intercurrent events only affecting one treatment group, because this action involves defining the outcome differently between treatments, which could introduce artificial differences
While-on-treatment/while-alive	The outcome before the occurrence of the intercurrent event is of interest	Modifies the endpoint attribute of the estimand
		Different while-on-treatment/while-alive estimands could be defined, depending on which outcomes are used before occurrence of the intercurrent event
		This strategy can compare outcomes at different time points between treatment groups, which can make the intervention appear effective (or harmful) even when it has no direct effect on the outcome
Hypothetical	The outcome pertaining to a hypothetical setting where the intercurrent event would not (or would) occur is of interest	Could modify the treatment attribute of the estimand
		Multiple hypothetical settings could apply, so the precise hypothetical setting envisaged should be described
		How the hypothetical setting would occur should be justified, to ensure that the estimand is well defined and to facilitate critical appraisal of the estimand’s clinical relevance
Principal stratum	The outcome in a subpopulation of patients who would not (or would) experience the intercurrent event is of interest	Modifies the population attribute of the estimand
		Different principal stratum populations can be defined—for instance, participants who would not discontinue either assigned treatment versus those who would not discontinue if assigned to intervention

### Treatment policy strategy

#### Definition

Under a treatment policy strategy, the occurrence of the intercurrent event is taken to be part of the treatment condition. For example, as part of assigning participants to a particular intervention, it is recognised that some participants will discontinue early, and interest lies in the effect of the intervention given it can lead to some early discontinuations. Thus, participant outcomes are used regardless of whether they experienced the intercurrent event or not.

#### Considerations for treatment-modifying events

The treatment policy strategy can be used to evaluate the effect of an intervention if it were used as part of routine practice, although only if the intercurrent event also occurs in practice. If not, use of a treatment policy strategy does not reflect conditions outside of the research setting.

#### Considerations for truncating events

Because the treatment policy strategy requires outcome data after the intercurrent event, it cannot be used for truncating events.

### Composite strategy

#### Definition

Under a composite strategy, the occurrence of the intercurrent event is incorporated into the endpoint definition, for instance, by assigning participants who experience the event a particular value of the outcome. The composite strategy modifies the endpoint attribute of the estimand. Different composite strategies can be used depending on which outcome value is assigned to participants (eg, in the example asthma study, participants who discontinue could be assigned a moderately abnormal FEV_1_ value of 60%, or a severely abnormal value of 50%). Each choice would correspond to a different estimand.

#### Considerations for treatment-modifying events

A composite strategy changes the interpretation of the endpoint, so care must be taken to ensure that the interpretation is not changed so much that it loses clinical relevance. For instance, if a composite strategy was used in the example asthma study, then the resulting treatment effect would not represent the mean difference in FEV_1_, but rather a mixture of the differences in both the discontinuation rates and FEV_1_ values, which might not be easy to interpret.

#### Considerations for truncating events

A composite strategy can be a useful way to ensure that death, or other truncating events, are reflected as poor outcomes. For example, in a covid-19 study, patients who die might experience fewer days on a ventilator; using a composite strategy to assign a poor value for patients who die (or alternatively, to redefine the outcome as days alive without a ventilator) ensures that death is not represented as a good outcome.^22^

### While-on-treatment/while-alive strategy

#### Definition

A while-on-treatment/while-alive strategy aims is to evaluate the effect of the intervention before the intercurrent event. Thus, only participant outcomes before the occurrence of the intercurrent event are used.^40^

The while-on-treatment strategy modifies the endpoint attribute of the estimand. Different while-on-treatment strategies can be defined. For instance, the outcome value immediately before the intercurrent event could be used. An alternative would be to use the average of the outcome across all time points before the intercurrent event.^41^ Terminology of the while-on-treatment strategy depends on the intercurrent event. If the intercurrent event is death, it is referred to as a while-alive strategy.

#### Considerations for treatment-modifying events

The while-on-treatment strategy can only be used when outcome data are available before the occurrence of the intercurrent event. Thus, it is well suited to binary outcomes that can be redefined as occurrence of the clinical event before the end of follow-up or the intercurrent event, whichever occurs first, or continuous outcome measures that are frequently measured across different time points.

This strategy can compare outcomes at different time points between intervention and control. In the example asthma study, if FEV_1_ scores become worse over time irrespective of treatment, and dupilumab leads to higher rates of early discontinuation, then a while-on-treatment strategy might demonstrate a beneficial effect for dupilumab just because early FEV_1_ values are used more frequently in the dupilumab group than in the placebo group. Thus, results should be interpreted in the light of any differences in intercurrent event rates between treatments.

#### Considerations for truncating events

The while-alive strategy can be used for truncating events such as death, when interest lies in what happened to the patient while they were still alive. For example, in palliative care or cancer studies, it might be useful to understand how treatment affected patients’ quality of life up to their death. However, the considerations listed above still apply, and so results should be interpreted in the light of any differences in rates of death between treatments.

### Hypothetical strategy

#### Definition

Under a hypothetical strategy, a hypothetical scenario is envisaged in which the intercurrent event would not (or would) occur, and participant outcomes corresponding to this hypothetical scenario are used. The aim is to evaluate the treatment effect in this hypothetical setting (eg, what the treatment effect would have been had the patients continued to take treatment). In the example asthma trial, if participants stopped dupilumab early because it was causing mild headaches, the hypothetical setting of interest might be if participants had instead continued dupilumab with the help of a mild analgesic to manage their headaches.

The hypothetical strategy can modify the treatment attribute of the estimand. For instance, in the hypothetical setting where participants do not discontinue dupilumab, the treatment attribute is changed to evaluate dupilumab under hypothetical compliance.

#### Considerations for treatment-modifying events

Researchers should define the mechanism used to avoid the intercurrent event in the hypothetical setting, because without such a mechanism the estimand is not well defined, and it would be impossible to know what participant outcomes ought to be. For instance, in the example asthma study, a hypothetical setting where participants are given analgesic to help them continue with dupilumab might lead to different outcomes compared with a setting where a lower dose of dupilumab is used that does not cause headaches, or compared with those from a setting where clinicians continue to use dupilumab despite its adverse effects. Definition of the mechanism also facilitates critical appraisal of the clinical relevance of the estimand. For instance, a research question centred around clinicians continuing to use a treatment despite adverse effects is unlikely to be clinically meaningful.

For these reasons, the hypothetical strategy will usually be most appropriate for intercurrent events that are subject to modification, because the mechanism behind the hypothetical scenario can usually be well defined.

#### Considerations for truncating events

As above, the mechanism behind the hypothetical setting should be defined. However, because truncating events such as death are usually not subject to modification (ie, we cannot specify how patients in a cancer study will avoid death), an appropriate mechanism cannot usually be defined.

### Principal stratum strategy

#### Definition

Under a principal stratum strategy, the estimand population is redefined to include only patients who would not (or would) experience the intercurrent event. The principal stratum strategy modifies the population attribute of the estimand. Different principal stratum populations can be defined. For instance, in the example asthma trial, the population could be defined as patients who would not discontinue early if assigned to either dupilumab or placebo. Conversely, it could be defined as those participants who would not discontinue early if assigned to dupilumab, regardless of whether they actually were assigned to dupilumab. For treatment-modifying intercurrent events, use of a principal stratum strategy is sometimes known as a complier average causal effect, whereas for truncating events such as death, it is often known as a survivor average causal effect.

#### Considerations for treatment-modifying events

In practice, we cannot determine which patients belong to the principal stratum population at the point they are assigned a treatment, because this information would require knowing their future intercurrent event status under each treatment strategy. Thus, if principal stratum effects are used to inform clinical decision making, some patients outside the principal stratum population might be treated on the basis of this result. Care should therefore be taken to ensure that treatment does not cause harm to those individuals outside this population. For instance, if dupilumab were to cause most patients to discontinue early owing to severe side effects, but use of the drug increases FEV_1_ in a small subset who can tolerate it, a principal stratum estimand would show a positive effect, which could result in most patients who are treated experiencing severe side effects.

#### Considerations for truncating events

The considerations outlined above also apply to truncating events. Further, for events such as death, a principal stratum strategy implies that outcomes before death (eg, a participant’s quality of life while they are alive) are irrelevant to the research question.

## Choice of estimand

The estimand should be chosen in line with the overall study objectives. For instance, if the aim is to evaluate the effect of the intervention as used in real world, routine clinical practice, the estimand should reflect this. This decision will require thinking through the potential intercurrent events that might occur and then identifying which strategy to handle each intercurrent event best matches the overall objectives.

Choice of estimand will also need to consider the estimation strategy (described below), because some estimands can be more reliably estimated than others. For instance, some estimands might require strong, implausible assumptions in order to estimate, or they might lead to larger amounts of missing data than other choices.^42^ Thus, some trade-off might be required between a perfect estimand that cannot be reliably estimated and a good but imperfect estimand that can be reliably estimated. Thus, choosing the estimand requires an iterative procedure, which might be revisited after consideration of study methods. Ideally, the estimand should be chosen collaboratively among the different stakeholders, including healthcare professionals, statisticians, and patient representatives.^43^

## Aligning study methods with the estimand

### Study design

The study design can affect the ability to estimate the desired estimand. For example, placebo run-in trials require all participants to start out on placebo. Then, only participants who adhere to placebo are randomised to stay on placebo or switch to the intervention. This design facilitates simple estimation of the treatment effect in the subset of patients who would adhere to placebo if assigned (ie, uses a principal stratum strategy). However, choice of strategy to handle such non-adherence is restricted by design, so only the principal stratum strategy can be used.

In general, studies that aim to estimate an intervention’s effect if used as part of routine practice should be designed to limit the occurrence of intercurrent events that would not occur in practice. For example, if an experimental rescue treatment is not available routinely, it should not be made available to study participants, because doing so would lead to a treatment comparison that does not reflect usual practice. Conversely, these studies should not be designed to minimise the occurrence of intercurrent events that would occur in practice because this design can also lead to comparisons that do not reflect routine practice.^5^

### Data collection

Data collection has an essential role in determining which estimands can be estimated, and how reliably. Thus, at the study outset, researchers must identify what data are required to support estimation of each estimand and ensure that they are collected. For instance, a treatment policy strategy requires outcome data to be collected even after the occurrence of the intercurrent event,^5^ and while-on-treatment/while-alive strategies require outcome data to be collected before the occurrence of the intercurrent event. Similar considerations exist for estimation of hypothetical^44^ ^45^ ^46^ ^47^ ^48^ ^49^ and principal stratum strategies.^4^ ^32^ ^33^ ^44^ ^50^ ^51^ ^52^

### Estimation

The appropriate method of statistical analysis (the estimator^5^) depends on which strategies have been specified to handle each intercurrent event. A brief overview of different estimators is provided in table 5, alongside references to articles that provide a more detailed description of how to implement certain methods.

**Table 5:** Overview of estimation methods used for different intercurrent event strategies

Intercurrent event strategy	Description of estimation methods*
Treatment policy	Estimated by including participant outcomes in the analysis regardless of the occurrence of the intercurrent event.
Composite	Estimated by first modifying the endpoint to make a composite, then including this modified endpoint in the analysis.
While-on-treatment/while-alive	Estimated by first modifying the endpoint (eg, by using outcome data from before the intercurrent event in place of final outcome data), and then including this modified endpoint in the analysis.
	Outcome data after the intercurrent event should not be set to missing, because doing so can lead to some participants being excluded from the analysis, which can induce bias; or lead certain statistical models, such as mixed models for repeated measures or the Cox model, to implicitly impute outcome data after the intercurrent event. This implicit imputation would then estimate a hypothetical strategy.^20^ ^21^
Hypothetical	Different methods can be used to estimate the hypothetical strategy.
	A common approach is to set outcome data after intercurrent events as missing data, and then use a method (eg, inverse probability weighting, multiple imputation, or likelihood based analyses) to try and recreate what the missing outcome data would have been, had the intercurrent event not occurred.^46^-^49^
	Alternative methods, such as instrumental variables^44^ or g estimation, have also been described.^48^
	Estimation of the hypothetical strategy requires assumptions that cannot be tested using the study data. Different methods require different assumptions, so the most appropriate method might vary from study to study depending on which set of assumptions is most realistic.
Principal stratum	Different methods can be used to estimate the principal stratum strategy, each of which require different assumptions.^4^ ^32^ ^33^ ^44^ ^50^-^53^ Several references^44^ ^51^ ^52^ provide an overview.
	When the intercurrent event is not affected by treatment assignment (ie, there are no patients who would experience the event in one treatment arm but not the other arm), a simple approach is to exclude patients who experience the intercurrent event from the analysis.^4^
	Instrumental variables can be used in many settings when occurrence of the intercurrent event is affected by treatment assignment.^44^
	More complex methods are typically required when the intercurrent event is death—for instance, those events that incorporate baseline covariates to help identify the principal stratums^50^

* Descriptions assume no missing outcome data. When missing outcome data are missing, some strategies require additional considerations.^45^ ^54^ ^55^ ^56^ ^57^ ^58^ ^59^

In the absence of missing data, the treatment policy, composite, and while-on-treatment/while-alive strategies can be estimated from a randomised trial with minimal assumptions. Conversely, estimation of hypothetical or principal stratum strategies requires stronger assumptions, which cannot be verified using the study data.^4^ ^32^ ^33^ ^35^ ^44^ ^45^ ^48^ ^49^ ^50^ ^51^ ^52^ ^60^ ^61^ ^62^ ^63^ ^64^ ^65^ ^66^ ^67^ This need for stronger assumptions is because the required data (such as the participant’s outcome in the hypothetical setting of interest or whether they belong to the principal stratum population) are unknown, and so assumptions about what these data might be are required. Therefore, estimation of these strategies can sometimes be less reliable (ie, more prone to bias) than estimation of the first three intercurrent event strategies. In non-randomised studies or studies with missing data, estimation of all strategies will typically require additional assumptions—for instance, around confounding or the nature of the missing data.

### Sensitivity analyses

Many analyses make certain assumptions about the study data, and when these are not fulfilled, they might produce biased (ie, incorrect) estimates of the treatment effect.^5^ ^28^ ^31^ ^42^ Sensitivity analyses are used to evaluate the robustness of results to departures from these assumptions, in order to inform investigators and readers about the reliability of results. For instance, if sensitivity analyses show similar results to the main results, investigators can have more confidence in their conclusions. Importantly, sensitivity analyses must target the same estimand as the main analysis, because obtaining a different answer to a different question gives no indication about the robustness of the results.

## Example 1: Applying the estimands framework to the FLO-ELA trial

We now demonstrate how the estimands framework can be implemented using the FLO-ELA trial, described earlier.^1^ FLO-ELA was an open label, pragmatic trial comparing two methods of fluid delivery (cardiac output monitor *v* clinician judgment) in patients undergoing emergency bowel surgery. The primary outcome measure was the number of days that participants were alive and out of hospital, within 90 days of randomisation. We describe the different steps of the estimands framework below. For clarity, we have simplified several aspects of the trial.

### Choice of estimand

The aim of FLO-ELA was to evaluate the effect of a treatment algorithm using a cardiac output monitor (COM) if used as part of real life routine practice. Thus, choice of the estimand attributes and the strategies to handle intercurrent events should reflect real life practice.

Defining an estimand requires specifying the first four attributes (population, treatments, endpoint, summary measure), and then anticipating which intercurrent events are likely to occur and deciding which strategies will be used to handle each type of intercurrent event.

Likely intercurrent events in FLO-ELA were thought to be (1) surgery might be cancelled after randomisation for some participants, either because they become too unwell or the underlying issue has resolved itself; (2) participants in the usual care group might be treated with the COM; (3) participants in the intervention group might not be treated with the COM; and (4) the COM might be used incorrectly (ie, the algorithm not followed).

A treatment policy strategy was chosen for intercurrent events 3 and 4 above (ie, the COM not being used, or being used incorrectly for intervention group participants); this strategy choice reflects that these intercurrent events could occur in practice and so can be considered an inherent part of the treatment.

Because current standard of care practice does not involve the use of a COM, allowing participants in the usual care group to use the COM does not reflect routine practice. Therefore, a hypothetical strategy, which envisions a setting where participants in the usual care group were not treated with the COM, would be most appropriate. However, this intercurrent event is likely to affect only a small handful of participants. Given the inherent challenges in estimating hypothetical strategies, a treatment policy strategy was chosen instead to simplify the analysis, under the assumption that it would have no material impact on results, given the low anticipated number of events.

As discussed earlier, although cancellation of surgery would occur in practice, a treatment policy strategy would not be appropriate as a means to evaluate the effect of the COM as used in practice. The COM can be used only for patients who undergo surgery, and so interest naturally lies in its effect in these patients. However, a treatment policy strategy would provide the effect of the COM regardless of whether patients underwent surgery or not. Thus, a principal stratum strategy based on the subset of patients who would undergo surgery, regardless of treatment assignment, is most appropriate. This strategy can also be easily estimated, as described below.^4^

Here, the handling of intercurrent events has affected the definition of the population attribute by clarifying that interest lies in patients who would undergo emergency bowel surgery under assignment to either treatment. It has also affected the treatment attribute, by clarifying that interest lies in the use of the COM regardless of whether it is used exactly as specified.

The full estimand is described in table 3, and can be written as: “The estimand for the primary outcome (DAOH90) is the ratio of means of days alive and out of hospital within 90 days of randomisation between protocolised, cardiac output guided, haemodynamic therapy versus usual care (intravenous fluid administered without use of cardiac output monitoring), regardless of adherence in the cardiac monitoring arm or use of cardiac monitoring in the control arm, in patients aged ≥50 years who would undergo emergency bowel surgery under assignment to either treatment.”

### Study design, data collection, and estimation

A standard, two arm, parallel group trial is sufficient to deal with the estimand in table 3. To ensure that the trial population was representative of the estimand population, specific recruitment strategies could be put into place to facilitate easier recruitment of under-represented groups, such as those presenting outside of normal working hours, and those lacking the capacity to consent (eg, owing to severe pain, or use of opioid analgesics).^1^ Ideally, the trial would also be designed to limit the number of enrolled participants who go on to have their surgery cancelled, for instance, by randomising participants as close to the start of surgery as possible. However, in practice a small delay between randomisation and surgery is inevitable, owing to the complexities involved in preparing the intervention.^4^

The outcome data required for estimation include outcomes even after the occurrence of intercurrent events for which a treatment policy strategy is being used.^5^ The occurrence of whether participants underwent surgery or not must also be collected to facilitate estimation of the principal stratum strategy.^4^

The estimand in table 3 can be estimated in a straightforward manner. The analysis population will be all randomised patients who did not have their surgery cancelled. Patients whose surgery was cancelled will be excluded from the analysis. This exclusion is to estimate the principal stratum strategy relating to the intercurrent event of cancellation of surgery^4^, which requires the assumption that cancellation of surgery is not affected by the treatment arm (ie, that patients who undergo surgery under the intervention arm would have also done so under the usual care arm, and vice versa).^4^ This assumption is justified on contextual grounds (ie, that it is implausible for a clinician to cancel surgery on the basis of the method of fluid delivery).^4^

### Sensitivity analyses

The main assumption underpinning the analysis described above relates to the approach to estimating the principal stratum effect, whereby participants who did not undergo surgery are excluded. The required assumption, described above, is justified on the contextual grounds, so formal sensitivity analyses are not required.^4^ If data are missing, the analysis would require additional assumptions, which would require sensitivity analyses (eg, to explore whether conclusions are affected under different assumptions around the missing data).^28^ ^68^

## Example 2: Applying the estimands framework to quality of life in a cancer trial

Investigators have developed a new pharmaceutical treatment for prostate cancer. They plan to run a pragmatic phase 3 trial to evaluate their new intervention against usual care, and expect it will lead to modest gains in overall survival of around three months. However, they are concerned that, owing to increased toxicity, the new intervention might reduce quality of life. Therefore, they wish to compare each patient’s average quality of life score (measured monthly) over one year between treatments so that patients and healthcare professionals understand the relative benefits and harms of the intervention.

### Choice of estimand

The trial objective is to evaluate the effect of the new intervention as used in routine practice and so intercurrent events (such as treatment discontinuation, missed doses, or switching to second line treatments) can all be handled using a treatment policy strategy. However, some patients will die before one year, so their quality of life scores are not defined past the point they die. Because a treatment policy strategy cannot be used for truncating events, investigators must decide which alternative strategy to use.

A hypothetical strategy considers the question “What would be the difference in the average quality of life over one year if men with prostate cancer never died?” However, this question does not match the trial objective, because the hypothetical setting considered does not match what happens in real life. Further, no mechanism to avoid death exists, and so the estimand itself is not well defined, meaning that any estimates produced by the trial will be challenging to interpret.

A principal stratum strategy considers the question “What is the difference in the average quality of life over one year in the subset of men who would survive past one year on either treatment?” As above, this question does not match the trial objective, because investigators are interested in the intervention’s impact on quality of life in all patients, even those who die.

Investigators next consider a composite strategy, where patients are assigned a quality of life score of 0 after they die. The investigators believe that this strategy broadly matches their objective, but are concerned that differences in quality of life due to the toxicity of the intervention might be obscured by its slightly lower incidence of the intercurrent event, and so results could be difficult to interpret.

Finally, the investigators consider a while-alive strategy, which looks at the question “What is the difference in the average quality of life over one year or until the patient has died, whichever is first?” Because this strategy includes quality of life scores from patients who die (ie, by using their average score before the point of death) it applies to all patients, and so investigators believe it matches their objective well. However, the investigators are concerned that any underlying time trends (eg, a reduction in quality of life over time, irrespective of treatment arm) might affect results, given the anticipated survival increase in the intervention arm.

After careful consideration, the investigators choose a while-alive strategy, because it best matches their objectives, and the strategy’s benefits outweigh its drawbacks. However, the investigators will be careful to interpret results in the light of any differences in mortality rates between treatment arms. The full estimand can then be written as: “The estimand is the difference in means of the average global quality of life score (measured monthly using the EORTC QLQ-C30) over one year or until death, whichever occurs first, between intervention plus usual care versus usual care alone, regardless of whether patients stop treatment early, switch to alternate treatments, or miss any treatment doses, in men aged ≥50 years with prostate cancer.”

### Study design, data collection, and estimation

Because a while-alive strategy requires outcome data before the intercurrent event, investigators plan to collect quality of life scores weekly for the first four weeks, then monthly thereafter.

Estimation is straightforward. The outcome is calculated by taking the mean of each patient’s quality of life scores over one year, or until the point they died, and the difference between arms can be estimated by including all randomised patients (even those with other intercurrent events, such as treatment discontinuation or switching) in a regression model. Importantly, methods that implicitly impute outcome data, such as mixed models for repeated measures, should not be used (table 5).

### Sensitivity analyses

In the absence of missing data, the estimator described above does not require any strong assumptions. If some data are missing, the estimator will require assumptions about the nature of the missing data (eg, missing at random), and so sensitivity analyses could be used to assess whether conclusions change under different assumptions.^28^ ^68^

## Discussion

Understanding the exact research question being answered in a study is essential for an appropriate interpretation of results. But most studies do not clearly define the research question, even when investigators attempt to describe it using existing frameworks, such as labelling the study as pragmatic or explanatory, or using the PICO (population, intervention, comparator, outcome) framework. This lack of definition is because these frameworks leave out key information essential to the proper interpretation of the research question.

The estimands framework resolves these problems by extending the PICO framework to include additional essential attributes. Estimands can therefore be used to clarify the exact interpretation of research questions by requiring investigators to describe each attribute of the treatment effect(s) they wish to quantify. By ensuring research questions are clearly described, estimands can help external stakeholders make informed decisions about interventions, by avoiding misinterpretations of study results. Estimands can also help study investigators to make sure they are using appropriate methods in their study relative to the research question they have chosen.

In this article, we have described the estimands framework outlined in the ICH E9(R1) addendum, which is now adopted by medicines regulators worldwide.^13^ However, other frameworks for describing treatment effects exist.^9^ ^34^ While the structure provided by the estimands framework is useful, the most important thing is to ensure the research question is described in sufficient detail to allow others to understand what the study is trying to estimate, regardless of the specific framework used.

## Notes

Provenance and peer review: Not commissioned; externally peer reviewed.
